# The Use of patient Reported Outcome Measures for Rheumatoid Arthritis in Japan: A Systematic Literature Review

**DOI:** 10.2174/1874312901711010043

**Published:** 2017-04-28

**Authors:** Ann Chuo Tang, Hyunchung Kim, Bruce Crawford, Taeko Ishii, Tamas Treuer

**Affiliations:** 1 Akasaka Garden City, 4-15-1 Akasaka, Minato-ku, Tokyo, 107-0052 Japan; 2 IMS Japan K.K., Toranomon Tower Office, 4-1-28 Toranomon, Minato-ku, Toyko, 105-0001 Japan; 3 Eli Lilly & Company Hungary. Lilly Hungária Kft. 1075 Budapest, Madách u. 13-14. (VII. emelet). Hungary

**Keywords:** Burden of illness, Japan, Patient-reported outcomes, QOL, Rheumatoid arthritis, Humanistic burden

## Abstract

**Background:**

Patient-reported outcomes (PRO) obtained through routine medical care may identify patients’ day-to-day burden and help tackle the disease from the patients’ perspective. However, there is a paucity of information regarding the availability of PRO data and PRO tools for rheumatoid arthritis (RA) in Japan.

**Objective:**

We reviewed the literature on PRO data availability and to identify PRO measures implemented in Japan for RA patients.

**Method:**

We conducted a systematic literature review using ICHUSHI and the PubMed databases on PRO measures for RA published from January 2011 to August 2015 in Japan.

**Results:**

After removing duplicates, 2423 manuscripts were found. From these, 100 manuscripts were included for review and analysis. We found 29 PRO tools that were used to assess various domains of health such as general well-being, pain, functionality, and fatigue. More than 90% of the studies utilized PRO tools for research purpose. Only one study reported PRO tool implementation in the routine medical care.

**Conclusion:**

The importance of PROs is recognized in Japan. PRO tools varied significantly and were mostly used for research purposes, while reports on the use of PRO measures in routine medical care were limited. Despite the awareness of PROs in the research community, unmet needs remain among RA patients in Japan. Further work is needed to investigate ways in which PROs can better reflect these unmet needs and be utilized in routine medical care.

## INTRODUCTION

Rheumatoid arthritis [RA] is a chronic autoimmune disease. Major features of the disease include systemic inflammatory polyarthritis causing pain, joint deformity and functional disability. The prevalence of RA is between 0.6% and 1.0% in Japan, affecting approximately 1.24 million Japanese patients. [1] RA negatively impacts patients’ ability to perform daily activities and their health related quality of life [HRQoL] [2, 3]. The functional disability and bodily pain are associated with mental well-being. Reports showed that 10-13% of RA patients are reported to suffer from depression [4-6]. Patients whose hobbies or social activities were negatively affected due to RA symptoms showed increased rate of depression in the following years [7, 8]. Furthermore, RA is associated with poorer sleep quality and increased physical fatigue [9, 10]. Thus, evaluating the disease from patients’ perspectives by using patient-reported outcomes [PROs] to assess pain, functional capacity, and fatigue *etc*. are essential in investigating the burden of RA [11, 12]. The impact of RA on patients and society is acknowledged in Japan [13] and there is growing awareness of HRQoL for RA patients. On the national level, the Ministry of Health, Labour, and Welfare [MHLW] established a rheumatism research committee in 2001 to investigate the disease, aiming for better disease control and delivery of accurate disease information to patients and clinicians [14]. As a part of this effort, the National Database of Rheumatic Diseases by iR-net in Japan [NinJa] was established in 2002. It contains clinical information and PROs of 11,940 RA patients. The therapeutic goals of RA have now encompassed not only clinical remission but also functional remission and sustainment of good HRQoL. The current treat-to-target guideline, which is highly utilized both globally and in Japan, lists “maximization of long-term HRQoL” as the primary treatment goal. The Japan Rheumatism Information Center, MHLW, Japan College of Rheumatology [JCR] and the Japanese RA patient advocacy group list maintenance of HRQoL as one of the RA treatment goals [15, 16].

Monitoring PROs in routine medical care could guide treatment choices for health care professionals when there is a discrepancy between patient and physicians’ assessment [12-17, 18]. Despite the increasing importance of patients’ HRQoL, currently there is no specific Japan guidance on how to measure HRQoL or what aspect of HRQoL to measure in the routine clinical setting. In addition, there is a lack of comprehensive literature review on PROs in the Japanese RA population to better guide Japanese PRO monitoring. Although RA burdens such as economic consequences are equally as important, this review focuses on reporting of PROs in Japan by addressing the following objectives: [1] to review the literature on RA specific PRO measures implemented in Japan and [2] to identify the future research needs on PRO measures for RA patients in Japan.

## METHODS

The review was conducted according to the Cochrane collaboration guidelines [19]. A Japanese language search of the Japan Medical Abstracts Society [ICHUSHI] database was conducted to identify articles from January 1, 2011 to August 14, 2015. An English language search of the MEDLINE [PubMed] database was conducted to identify articles published from January 1, 2014 to August 14^th^, 2015 to capture the most recent publication. The search terms used for PubMed and ICHUSHI are presented in Appendix A and B. In order to identify additional articles that were not listed in PubMed and ICHUSHI, a review of the grey literature was conducted on the following websites: Japan Rheumatism Foundation Information Center, ACR, JCR, MHLW, Outcome Measures in Rheumatology Clinical Trials [OMERACT], and the Japanese RA patient advocacy group “Japan Rheumatism Friendship Association”. The inclusion criteria comprised articles reporting PROs of RA in Japanese adult population living in Japan. Articles were excluded if they were duplicates, not in English or Japanese languages, not a country of interest or if abstracts were unavailable for review. Furthermore, letters, editorials, commentaries, case studies, and animal models/studies were not included. Two reviewers conducted a title/abstract review followed by a full-text review to select and appraise relevant studies. Disagreements were resolved through discussions and results were checked by a third reviewer. Data including year of publication, objectives, data source, study design, PRO results and utilized PRO measures were extracted prior to data synthesis. PRO results were further categorized by a number of domains of health, including general well-being, pain, functionality, psychological status, fatigue, morning stiffness, and other domains.

## RESULTS

Based on the inclusion and exclusion criteria, 100 articles were selected for in-depth analysis Fig. (**1**). Among 100 studies investigating PROs in patients with RA in Japan, 2 studies were clinical trials, and 98 studies were observational studies. [Full list available as supplementary material]

### Awareness of PROs in Japan

A number of studies recognized the importance of assessing not only the clinical outcomes of RA but also outcomes that have significant impact on patients [20-25]. Despite this recognition, most of the identified literature assessed PROs for research purposes only and did not specify the use of PRO measures in routine medical care. Only one study reported the use of PROs in a daily medical setting [26]. This cross-sectional study found less than 10% of RA patients treated by rheumatologists were evaluated with Health Assessment Questionnaire [HAQ] or modified HAQ [mHAQ]. Patient global assessment [PtGA] using the visual analogue scale [VAS] was implemented in 43.0% of the RA patients [26] but the details of the PtGA were not reported. The PRO tools utilization by non-rheumatologist clinicians was even more infrequent where HAQ and mHAQ were used in less than 1% of the patients and PtGA was used in 12.9% of the patients [26].

### Measurement Tools Used to Assess PROs Among RA in Japan

**Fig. (3):**
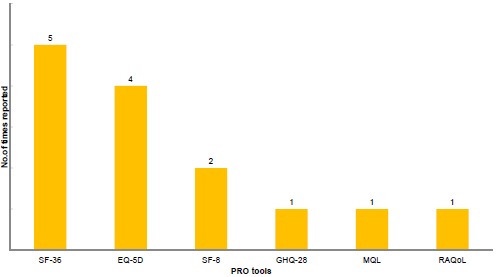
SF-36: Medical Outcome Study Short Form Health Survey; EQ-5D: EuroQol 5 Dimension; SF-8: Medical Outcome Study Short Form -8; GHQ-28: General Health Questionnaire-28; MQL10: Mokichi Okada Association QOL questionnaire; RAQoL: Rheumatoid Arthritis Quality of Life Questionnaire.

We found 29 PRO tools used in the Japanese studies but many of them were used only once, showing an inconsistency of PRO tool utilization in Japan Fig. (**2**). The 29 tools measured a variety of health domains, including general well-being, fatigue, morning stiffness, psychological status, pain, and physical function. The most frequently implemented tools [used in more than 10 studies] included those recommended in the original OMERACT RA core set for clinical trials. The common tools were for the assessment of pain, function, and disease activity [27]. Most of the studies were observational studies investigating the impact of treatments in patients’ well-being, and few studies investigated the HRQoL of patients with RA in general (Fig. **3**). The pain VAS was the main pain measurement tool utilized in 21 out of 23 Japanese studies reporting pain-related outcomes [91.3%]. Twenty one observational studies reported pain related outcomes. Pain intensity was assessed following treatments or was measured *via* cross-sectional surveys to explore the relationship between pain and other PROs. Pain was generally reported to strongly correlate with functional disability and anxiety. [20, 25] Some studies explored pain through qualitative interviews [*e.g.* “what was the most excruciating pain you’ve experienced when conducting your daily life activities”, “throughout the year when does it hurt the most”, *etc.*] [28, 29].

**Fig. (4):**
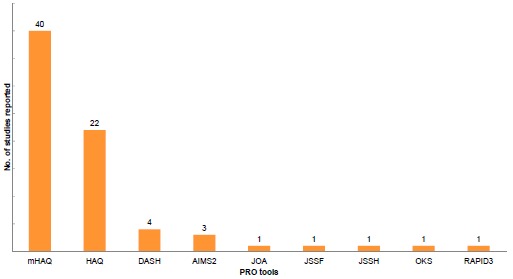
Distribution of functional PRO tools utilization.

Physical function was measured in more than half of all studies reviewed. The mHAQ was the most frequently implemented functional assessment tool, followed by the HAQ. Most (38 of studies on physical function) studies merely reported the change in HAQ or mHAQ scores, without further investigation of other PROs. Other functional assessment tools included the Disabilities of the Arm, Shoulder, and Hand (DASH), Arthritis Impact Measurement Scales [AIMS-2], Routine Assessment of Patient Index [RAPID 3], and tools measuring functions of specific locations of the body (*e.g.* hand, wrist, knee, foot) (Fig. **4**).

**Fig. 5:**
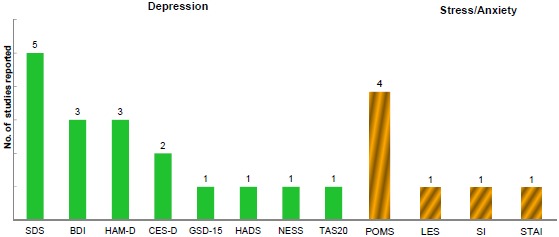
Distribution of psychological status PRO tools utilization.

PROs related to patients’ psychological status such as depression, anger, stress, and anxiety were assessed. All of the psychological related studies were observational studies, either investigating patients’ current mental HRQoL after being diagnosed with RA or the change in psychological status following treatment interventions. Eight PRO tools were utilized to measure depression Fig. (**5**). The Zung Self-rating Depression Scale [SDS] was the most frequently reported tool in Japan, followed by the Beck Depression Inventory [BDI-2] and Hamilton Depression Rating Scale [HAM-D]. Although depression was measured among RA, the literature suggests that an appropriate PRO tool for depression screening would be needed as depression because chronic RA is difficult to detect and treat [25]. Stress and anxiety were measured using four stress- and anxiety-specific PRO measures, with the Profile of Mood State (POMS) being the most frequently utilized tool. (Fig. **5**).

Overall, there was a paucity of Japanese articles reporting morning stiffness. Two studies reported the duration of morning stiffness, but did not assess severity or its impact on the patients’ life [30, 31].

Fatigue was reported in five observational studies, with four studies using a composite index of the POMS. One study investigated the fatigue level of both biologics users and biologics-naïve patients in regards to difficulties of convalescence from RA at home. The results showed that fatigue was one of the top difficulties during convalescence in both cohorts [32].

Patients’ perception of illness was frequently evaluated using the PtGA for disease status or through qualitative studies. One study reported caregiver burden and its correlation to patients’ reported burden using the Zarit caregiver burden index. Caregivers with disease were reported to have higher depression values than caregivers without any disease [33].

## DISCUSSION

We reviewed the utilization of PRO tools for RA patients in Japan. To the authors’ knowledge, this is the first systematic review that focused on the use of PRO measures for the Japanese RA population. Among the 100 studies reviewed, we found that, 1) the value of PROs were recognized in the Japanese RA research community, but did not demonstrate the widespread use of PRO tools in the clinical setting, 2) a number of PRO tools were used but mainly for specific research needs, 3) there was heterogeneity of PRO tool utilization, and 4) PROs measured mainly focused on physical function and pain.

Shimizu and Minami highlighted the importance of collecting comprehensive patient information on psychological, economic, and daily life impact [22, 24]. Takahashi mentioned that PROs may contribute to better communicate between clinicians and patients, and guide treatment decisions [34]. PROs play a critical role in determining treatment pathways and timing of treatment [35]. Tight control of RA and close monitoring using PROs between the timeframe of onset to joint destruction, also known as the “window of opportunity”, were considered to contribute to having a higher chance of achieving clinical remission and prevent joint damage [36]. While the importance of PRO is being recognized, the more common understanding of PRO seems to be focused on functionality. Increasing evidence shows that functionality alone does not predict better outcomes. The ACR guidelines note that high HAQ score is a poor prognostic factor for joint damage [35]. More studies to show how measurements of PROs may contribute to better patient care on both clinical and patient level would improve patient-focused holistic care.

Our review demonstrated that despite a number of studies reporting PRO tools, except for Takeuchi’s study, there was a lack of studies reporting the use of PRO tools in routine medical care. This may imply that PRO tools are generally underutilized in Japanese routine clinical setting. There are several possible reasons behind the underutilization of PROs in clinical settings in Japan. First, there is a general lack of consensus and guidance on the use of PROs in daily medical care. The current Japanese clinical guideline does not indicate a specific PRO to address nor recommend the use of a specific PRO tool in any of the treatment recommendations. The treat-to-target guideline, which is highly utilized both globally and in Japan, emphasizes the use of validated composite measures of disease activity. Most of the parameters used are to support disease activity assessment rather than to measure humanistic burden [37]. Second, the unavailability of good PRO tools to accommodate the limited time in a busy medical setting may be another reason why PROs are not actively utilized [24]. There are many PRO tools used outside of Japan that have been evaluated for validity, reliability, sensitivity to change, and feasibility and these tools are not yet validated in Japan. Identifying appropriate measures and performing psychometric validation studies in the Japanese context would be valuable.

From the review we conducted, we found a significant inconsistency in the PRO tool utilization and most of the tools were only implemented once. Out of the 8 depression-related PRO measures, 6 were used once, and out of the 11 function related tools, 7 tools were used once. This result was consistent with Kalyoncu’s review conducted outside of Japan. They also concluded the heterogeneity of PRO tool utilization where 63 PRO tools were used to measure 14 domains of health in 109 RA reports [11]. Considering the complexity and inconsistency of the use of PROs in RA, medical education regarding proper selection and use of appropriate PROs to encourage utilization of PRO tools in clinical settings may be beneficial.

Our result showed that the number of research on physical functions outweighs the others. This may imply that the current RA treatment is more focused on functionality and disease activities. It may also suggest the need to develop an easy-to-use, clinically meaningful and relevant PRO tool in daily practice. Other than functionality, outcomes such as sleep disturbance, fatigue, and morning stiffness in Japan are recognized as an unmet need and are captured through the use of specific PRO tools in countries outside of Japan [38-43]. Medical Outcomes Study-sleep questionnaire [MOS] and Pittsburg Sleep Quality Index [PSQI] were developed outside of Japan. However, no studies measuring sleep disturbance or sleep quality in Japanese RA patients were found in this review. As for fatigue, a number of articles were identified using fatigue specific assessment tools such as the Functional Assessment of Chronic Illness Therapy- Fatigue [FACIT-fatigue] and Fatigue Severity Scale [FSS] from abroad. One literature review indicated that fatigue can be caused by the use of MTX, which is a standard RA treatment option in many countries including Japan [44]. Research that differentiates treatment-induced versus disease-induced fatigue is warranted. Morning stiffness was recognized as an unmet treatment need as currently there are no pharmacological treatments that can ease the symptoms but it has been persistently reported by patients [45]. For morning stiffness, only 2 studies identified measuring the duration of morning stiffness. On the other hand, morning stiffness could be measured multi-dimensionally [*i.e.* presence of morning stiffness in certain timeframe, severity of morning stiffness and intensity of morning pain]. Given that these specific symptoms are often not captured in tools that measure overall functionality or general HRQoL, development of a new tool which is sensitive enough to capture these concepts is needed in the future.

Pain was measured in a majority of the articles using the VAS. Other validated PRO tools were not identified except for some qualitative studies investigating various types of pain [*i.e.* pain due to weather or certain time of the day] [28]. Pain is a multidimensional concept intertwining not only intensity but also quality, location, duration, and the psychological impact. Studies from abroad have used pain related tools to measure RA pain from various perspectives. For example, Joharatnam used the McGill Pain questionnaire and widespread pain index to measure pain by location and duration [46]. Lee used the Brief-Pain Inventory to measure the impact of RA pain on work, relations with other people, mood and sleep [43]. Further research in Japan using validated tools that can capture pain effectively would be warranted.

This study has a few limitations. First, the literature search was only conducted in PubMed and ICHUSHI. The other large biomedical database, EMBASE, was not used in this study. However, literature in EMBASE is mostly European focused and covers only 2% of the total Japanese literature. Therefore, most of the Japanese articles would have been captured by the ICHUSHI and PubMed database. Second, the search timeframe for PubMed and ICHUSHI for this report was different. However, the conclusion derived from this review would not differ much in terms of the variation of PRO tools in Japan and patient-reported unmet needs in the Japanese real world setting, as similar trends were consistently demonstrated in studies outside of Japan for the past 10 years [11, 38, 47]. Finally, a review study may be subject to a product of publication bias. In the case of this review, the underuse of PROs in daily practice may not be engaging enough to warrant publication in some journals and therefore underreported.

## CONCLUSION

The importance of PROs is recognized in Japan. PRO tools identified varied significantly and were mostly used for research purposes, while reports on the use of PRO measures in routine medical care was limited. Despite the awareness of PROs in the RA research community, unmet needs remain among RA patients in Japan. Further work is needed to investigate ways in which PROs can better reflect these unmet needs and be utilized in routine medical care. Activities such as developing easy-to-use and clinically meaningful tools, education on and incorporation of PROs in specific guidelines would likely improve RA patient care in Japan.

## APPENDICES

### Appendix A: MEDLINE search Strategy

## SUPPORTIVE/SUPPLEMENTARY MATERIAL

1. List of included articles (n=100).

## Figures and Tables

**Fig. (1):**
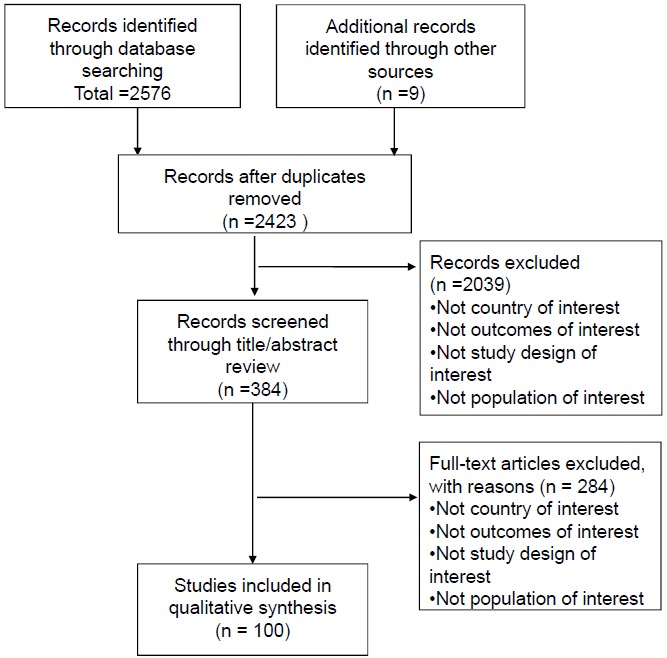
PRISMA flow diagram.

**Fig. (2):**
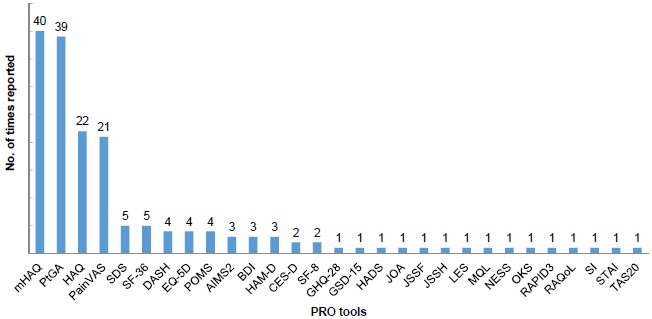
mHAQ: modified Health Assessment Questionnaire; PtGA: Patient Global Assessment; HAQ: Stanford Health Assessment Questionnaire; Pain VAS: Pain Visual Analogue Scale; SDS: Zung Self-rating Depression Scale; SF-36: Medical Outcome Study Short Form Health Survey; DASH: Disabilities of the arm, shoulder, and hand; EQ-5D: EuroQol 5 Dimension; POMS: Profile of Mood State; AIMS-2: Arthritis Impact Measurement Scale; BDI: Beck Depression Inventory; HAM-D: Hamilton Depression Rating Scale; CES-D: Center for Epidemiologic Studies- Depression; SF-8: Short form-8; GHQ-28: General Health Questionnaire-28; GSD-15: Geriatric Depression Scale; HADS: Hospital Anxiety Depression Scale; JOA: Japanese Orthopedic Association score; JSSF: Japanese society for surgery of the foot; JSSH: Japan Society for Surgery of the Hand Score of ADL; LES: Life Experiences Survey; MQL10: Mokichi Okada association QOL questionnaire; NESS: Negative Emotional Suppression Scale; OKS: Oxford Knee Score; RAPID3: Routine Assessment of Patient Index Data 3; RAQoL: Rheumatoid Arthritis Quality of Life; SI: Stress Inventory; STAI: State-Trait Anxiety Inventory; TAS-20: Toronto Alexithymia Scale.

**Appendix B:** ICHUSHI search strategy.

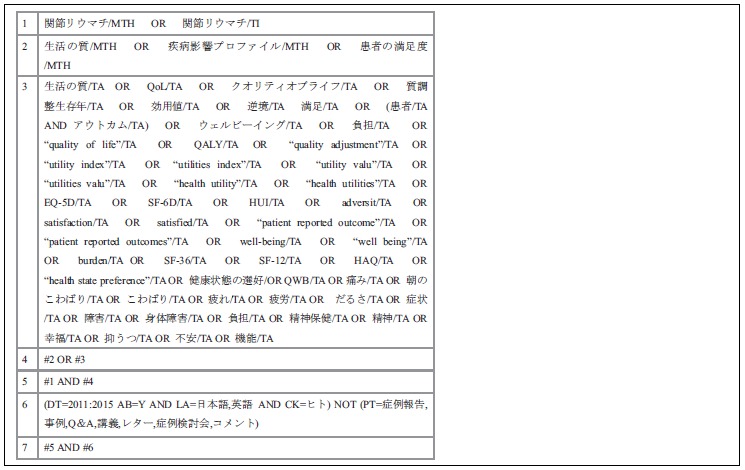

**Table:** 

1	“Arthritis, Rheumatoid”[Majr] OR rheumatoid arthritis[Title]
2	“Quality of life”[Majr] OR “Sickness Impact Profile”[Majr] OR “Patient Satisfaction”[Majr]
3	quality of life[tiab] OR QALY[tiab] OR quality adjustment[tiab] OR utility index[tiab] OR utilities index[tiab] OR utility valu*[tiab] OR utilities valu*[tiab] OR health utility[tiab] OR health utilities[tiab] OR EQ-5D[tiab] OR SF-6D[tiab] OR adversit*[tiab] OR satisfaction[tiab] OR satisfied[tiab] OR patient reported outcome[tiab] OR patient reported outcomes[tiab] OR well-being[tiab] OR well being[tiab] OR burden[tiab] OR SF-36[tiab] OR SF-12[tiab] OR HAQ[tiab] OR prefer*[tiab] OR QWB[tiab] OR pain[tiab] OR “morning stiffness”[tiab] OR tiredness[tiab] OR fatigue[tiab] OR symptom*[tiab] OR dysfunction[tiab] OR disability[tiab] OR “mental health”[tiab] OR happiness[tiab] OR depress*[tiab] OR anxiety[tiab] OR function*[tiab] OR “Health Assessment Questionnaire”[tiab]
4	fatigue[Majr] OR Depression[Majr] OR Mental health[Majr] OR Pain[Majr] OR Anxiety[Majr] OR Quality-Adjusted Life Years[Majr] OR Patient Preference[Majr] OR Symptom Assessment [Majr]
5	#2 OR #4
6	#5 AND #1
7	#3 AND #1
8	#6 OR #7
9	(“2014/01/01”[PDAT]: “2015/8/14”[PDAT]) AND English[lang]
10	#8 AND #9
